# Downregulation of angulin-1/LSR induces malignancy via upregulation of EGF-dependent claudin-2 and TGF-β-dependent cell metabolism in human lung adenocarcinoma A549 cells

**DOI:** 10.18632/oncotarget.27728

**Published:** 2023-03-24

**Authors:** Wataru Arai, Takumi Konno, Takayuki Kohno, Yuki Kodera, Mitsuhiro Tsujiwaki, Yuma Shindo, Hirofumi Chiba, Masahiro Miyajima, Yuji Sakuma, Atsushi Watanabe, Takashi Kojima

**Affiliations:** ^1^Department of Thoracic Surgery, Sapporo Medical University School of Medicine, Sapporo, Japan; ^2^Department of Cell Science, Research Institute for Frontier Medicine, Sapporo Medical University School of Medicine, Sapporo, Japan; ^3^Department of Respiratory Medicine and Allergology, Sapporo Medical University School of Medicine, Sapporo, Japan; ^4^Department of Pathology, Sapporo Medical University School of Medicine, Sapporo, Japan; ^5^Department of Molecular Medicine, Research Institute for Frontier Medicine, Sapporo Medical University School of Medicine, Sapporo, Japan

**Keywords:** angulin-1/LSR, claudin-2, cell metabolism, malignancy, lung adenocarcinoma

## Abstract

Abnormal expression of bicellular tight junction claudins, including claudin-2 are observed during carcinogenesis in human lung adenocarcinoma. However, little is known about the role of tricellular tight junction molecule angulin-1/lipolysis-stimulated lipoprotein receptor (LSR). In the lung adenocarcinoma tissues examined in the present study, expression of claudin-2 was higher than in normal lung tissues, while angulin-1/LSR was poorly or faintly expressed. We investigated how loss of angulin-1/LSR affects the malignancy of lung adenocarcinoma cell line A549 and normal human lung epithelial (HLE) cells. The EGF receptor tyrosine kinase inhibitor AG1478 prevented the increase of claudin-2 expression induced by EGF in A549 cells. Knockdown of LSR induced expression of claudin-2 at the protein and mRNA levels and AG1478 prevented the upregulation of claudin-2 in A549 cells. Knockdown of LSR induced cell proliferation, cell migration and cell metabolism in A549 cells. Knockdown of claudin-2 inhibited the cell proliferation but did not affect the cell migration or cell metabolism of A549 cells. The TGF-β type I receptor inhibitor EW-7197 prevented the decrease of LSR and claudin-2 induced by TGF-β1 in A549 cells and 2D culture of normal HLE cells. EW-7197 prevented the increase of cell migration and cell metabolism induced by TGF-β1 in A549 cells. EW-7197 prevented the increase of epithelial permeability of FITC-4kD dextran induced by TGF-β1 in 2.5D culture of normal HLE cells. In conclusion, downregulation of angulin-1/LSR induces malignancy via EGF-dependent claudin-2 and TGF-β-dependent cell metabolism in human lung adenocarcinoma.

## INTRODUCTION

Lung cancer is the most common cause of cancer death in the world, with an estimated ~1.6 million deaths each year [1]. About 85% of lung cancer patients have a group of histological subtypes collectively known as non-small cell lung cancers (NSCLC) in which lung adenocarcinoma and lung squamous cell carcinoma are the most common subtypes [2]. Although approximately 85% of all lung cancers are related to cigarette smoking, recent evidence suggests that the number of lung adenocarcinoma cases in nonsmokers is rising [2]. Alterations in key pathway components for receptor tyrosine kinase signaling, mTOR signaling, the oxidative stress response, proliferation and cell cycle progression are involved in NSCLC [3]. Patients (~15%) diagnosed with EGFR-mutant NSCLC have a good initial clinical response to epidermal growth factor receptor (EGFR) tyrosine kinase inhibitors (EGFR TKIs), but tumor recurrence is common and quick to develop. Accordingly, continued studies of new drugs and combination therapies are needed to improve NSCLC outcomes [3].

Tight junctions (TJ) are epithelial and endothelial cell-cell junctions that regulate the flow of solutes through paracellular pathways and maintain cell polarity, thereby functioning as a barrier of epithelial and endothelial cellular sheets [4]. Tricellular tight junctions (tTJs) form at the convergence of bicellular tight junctions (bTJs) where three epithelial cells meet in polarized epithelia [5, 6]. Several studies have reported that loss of bTJ proteins, including claudins (CLDNs) and occludin (OCLN), enhances tumor progression [7–10]. Upregulation of claudin-2 (CLDN-2) expression in human lung, liver, colon and stomach cancer tissues is reported [11]. CLDN-2 is highly expressed in lung adenocarcinoma tissues and increases proliferation in adenocarcinoma cells [12]. CLDN-2 expression is increased via an EGFR/MEK/ERK/c-Fos pathway in lung adenocarcinoma A549 cells [13]. Overexpression of CLDN-2 closely contributes to the malignancy of endometrioid endometrial adenocarcinoma [14]. Overexpression of CLDN-3 also promotes the malignancy of lung adenocarcinoma via EGF-activated MEK/ERK and PI3K/Akt pathways [15].

On the other hand, angulin-1/lipolysis-stimulated lipoprotein receptor (LSR) is a novel molecular constituent of tricellular contacts localized in most epithelial tissues and has a barrier function [4, 16]. Angulin-1/LSR recruits tricellulin (TRIC), which is the first molecular component of tTJs, and the interaction between the cytoplasmic domain of angulin-1/LSR and the C-terminal cytoplasmic domain of TRIC is required for this recruitment [4]. Loss of angulin-1/LSR affects the malignancy of various cancers, including bladder cancer, colon cancer, endometrial cancer, head and neck cancer and pancreatic cancer [18–21]. Furthermore, downregulation of angulin-1/LSR promotes cell invasion via upregulation of CLDN-1-mediated MMPs in endometrial cancer cells [19]. In pancreatic cancer, angulin-1/LSR contributes to the epithelial barrier and malignancy via the growth factors EGF and TGF-β [21]. Furthermore, knockdown of angulin-1/LSR increases the expression of CLDN-2 in airway epithelial Calu-3 cells [22]. However, the role and regulation of anguln-1/LSR in lung cancer remain unknown.

Lung adenocarcinoma with activated epidermal growth factor receptor (EGFR) mutations is dependent on EGFR signaling for survival and proliferation [23]. The EGFR tyrosine kinase inhibitor AG1478 inhibits the migration and invasion of human lung adenocarcinoma cells via cell cycle regulation by MMP-9 [24]. In A549 cells, the promoter activity of human CLDN-2 is decreased by AG1478 and it prevented the upregulation of CLDN-2 induced by EGF [13].

Several tumors, including lung adenocarcinoma, highly express transforming growth factor-β (TGF-β), which contributes to tumor progression [25]. TGF-β signaling promotes epithelial to mesenchymal transition (EMT), invasion and metastasis in lung adenocarcinoma [23]. It also alters the epithelial barrier with modification of claudins in various cells [26, 27]. The TGF-β1 induces EMT and epithelial permeability in respiratory epithelial cells [28]. TGF-β receptor type-1 inhibitor EW-7197 inhibits breast to lung metastasis [29]. EW-7197 prevents changes of the distribution and barrier function of angulin-1/LSR by TGF-β1 in pancreatic cancer [21]. It also prevents downregulation of angulin-1/LSR expression and the epithelial barrier, and upregulated CLDN-2 expression and cell metabolism induced by high mobility group protein B1 (HMGB1), a damage-associated molecular pattern (DAMP) protein, in bronchial adenocarcinoma cell line Calu-3 [22]. Overexpression of HMGB1 correlates with the proliferation and metastasis of lung adenocarcinoma [30]. EW-7197 is Vactosertib and there is a clinical trial (Phase 1b/2a, NCT03732274) in advanced NSCLC [31].

Metabolic reprogramming is recognized as one of the main hallmarks of cancer [32, 33]. TGF-β induces metabolic reprogramming during EMT in cancer [34]. Transcription factor Forkhead box protein M1 (FOXM1) promotes EMT via aberrant cell metabolism in pancreatic cancer [35] and it also enhances lung adenocarcinoma invasion and metastasis [36]. In the paclitaxel-resistant lung adenocarcinoma cell line A549/Taxol, alterations of cell metabolism indicated as mitochondrial morphology and functions are observed [37]. CLDN-2 knockdown decreases cell metabolism in human endometrial adenocarcinoma cell line Sawano [14]. It is thought that the altered metabolism in lung cancer is also a key for the diagnosis and has implications for the prognosis and response to treatments.

It is thought that distal airway stem cells in the lung can differentiate into bronchioles and alveoli [38]. We previously reported that p63-positive human lung epithelial (HLE) cells derived from normal human lung tissues can differentiate into bronchiole-like or alveolus-like organoids and that HLE cells undergo EMT [39]. Some p63-positive basal cells undergo EMT, and knockdown of p63 prevents the phenotypic switch in normal bronchial epithelial cells [40]. However, little is known about the detailed regulation of EMT from normal human peripheral lung epithelial cells.

In the present study, we investigated the role and regulation of tTJ protein angulin-1/LSR in the malignancy of human lung adenocarcinoma compared to normal lung epithelial cells. We found that downregulation of angulin-1/LSR induced malignancy via upregulation of EGF-dependent CLDN-2 and the TGF-β-dependent cell metabolism in human lung adenocarcinoma, and that both AG1478 and EW-7197 had potent *in vitro* anti-lung adenocarcinoma therapeutic activity via LSR/CLDN-2 and the cell metabolism.

## RESULTS

### Expression and localization of LSR and CLDN-2 in lung adenocarcinoma

**Figure 1:**
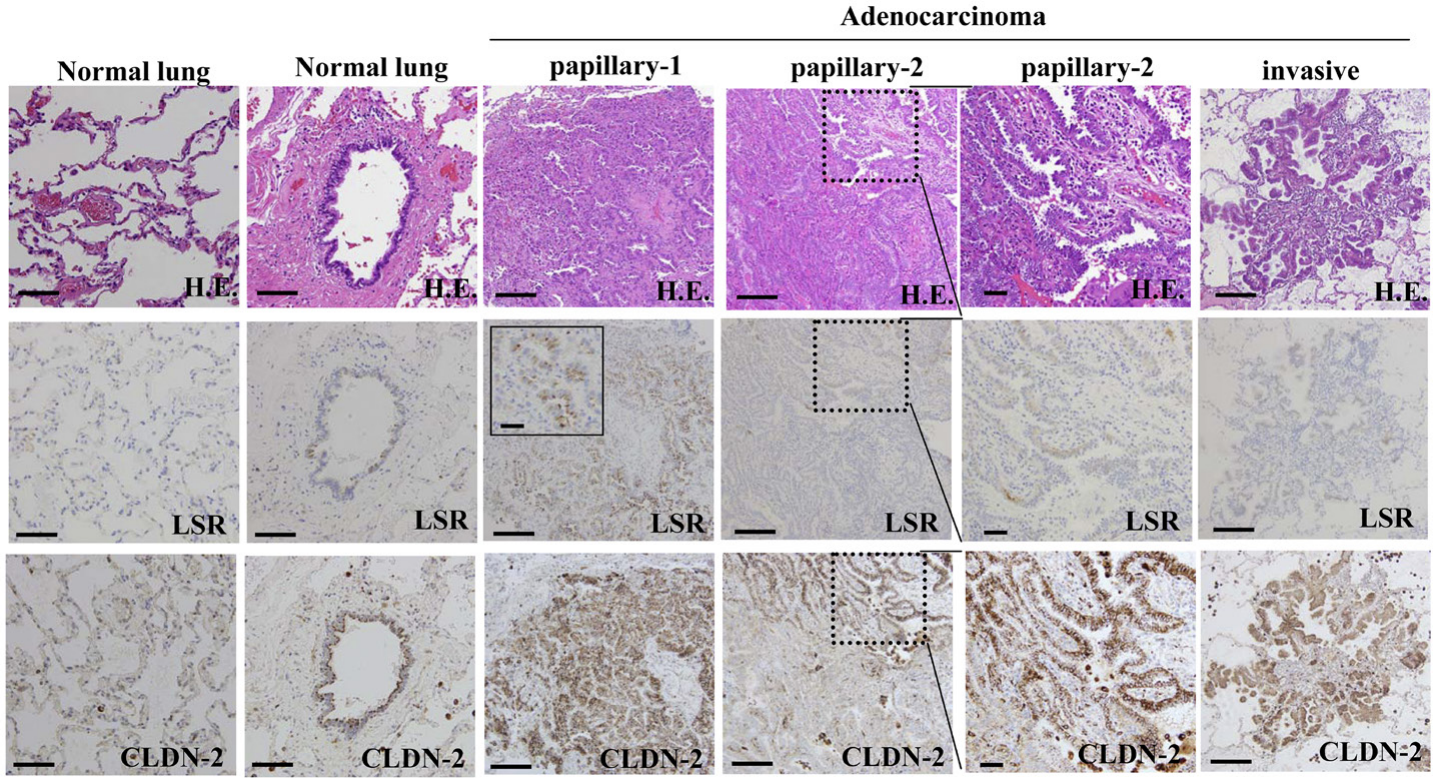
Expression and distribution of LSR and CLDN-2 in human lung adenocarcinoma. Hematoxylin and eosin (H. E.) and immunohistochemical staining for LSR and CLDN-2 in normal (peripheral bronchial, alveolar) and lung adenocarcinoma (papillary, invasive) tissue. Lined square of LSR in papillary-1 is a high magnification image. Dot lined squares in papillary-2 are areas to high magnification images. Scale bars: 100 μm.

To investigate changes in the distribution and expression of LSR and CLDN-2 during carcinogenesis of human lung adenocarcinoma, immunohistchemical staining for LSR and CLDN-2 was performed using paraffin-embedded sections of lung cancer tissues (8 different adenocarcinomas, 2 invasive, 3 papillary, 1 acinar, 2 solid). In normal lung tissues, LSR was faintly expressed and CLDN-2 was detected in peripheral bronchial epithelium, while LSR and CLDN-2 were not detected in alveolar epithelium (Figure 1). In one papillary adenocarcinoma, both LSR and CLDN-2 were highly expressed at the membranes and in the other adenocarcinomas. Only CLDN-2 was highly expressed at the membranes and in the cytoplasm, and LSR was faintly expressed at the membranes (Figure 1).

### Knockdown of LSR induced expression of CLDN-2 at protein and mRNA levels via EGF signaling in lung adenocarcinoma cell line A549

**Figure 2:**
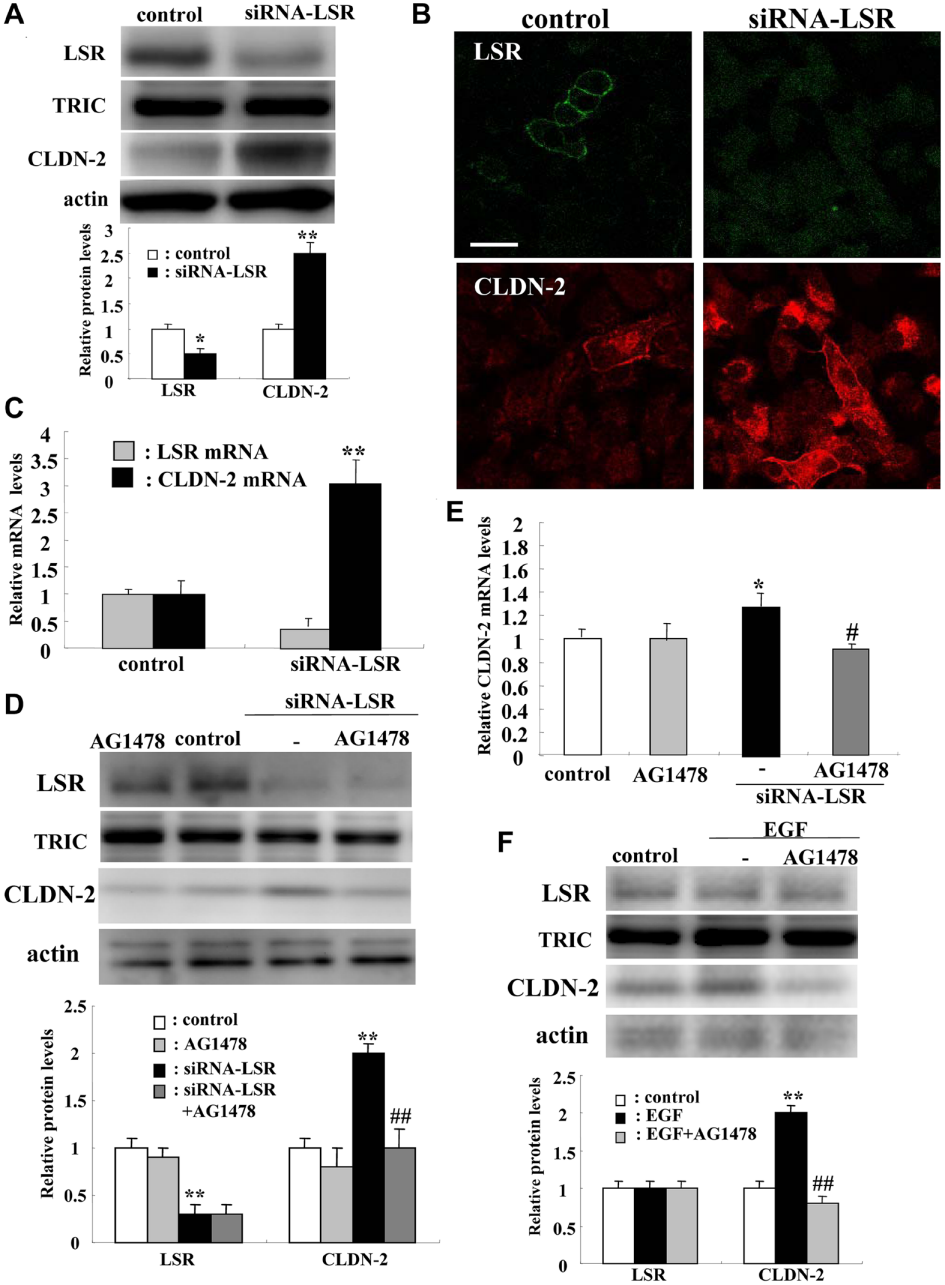
Knockdown of LSR induces CLDN-2 expression at mRNA and protein levels via EGFR signaling in human lung adenocarcinoma A549 cells. Western blotting (**A**), immunocytochemical staining (**B**) and real-time PCR (**C**) for LSR, TRIC, and CLDN-2 in A549 cells transfected with siRNA-LSR. Western blotting (**D**) and real-time PCR (**E**) for LSR, TRIC, and CLDN-2 in A549 cells transfected with siRNA-LSR with and without AG1478. Western blotting (**F**) for LSR, TRIC and CLDN-2 in A549 cells pretreated with AG1478 before treatment with EGF. The corresponding expression levels of (A, D, and F) are shown as bar graphs. ^**^ *p* < 0.01, vs control, ^*^ *p* < 0.05, vs control, ^#^ *p* < 0.05, vs TGF-β1.

We first used two human adenocarcinoma cell lines A549 and Calu-3. Although in Calu-3 cells, knockdown of LSR induced CLDN-2 expression similar to that of A549 cells, CLDN-2 expression was low level and the effects of TGF-β were not observed (Supplementary Figure 1). We used A549 cells as lung adenocarcinoma cells through all experiments. To investigate how knockdown of LSR induced malignancy, cell proliferation and migration in lung adenocarcinoma, we first performed DNA microarray analysis in lung adenocarcinoma A549 cells transfected with the siRNAs of LSR. In Western blotting and immunocytochemical staining, knockdown of LSR increased expression of CLDN-2 protein at the membranes and in the cytoplasm (Figure 2A and 2B). Real-time PCR analysis revealed that knockdown of LSR increased expression of CLDN-2 mRNA compared to the control (Figure 2C). Treatment with the tyrosine kinase inhibitor AG1478 inhibited the upregulation of CLDN-2 protein induced by knockdown of LSR, whereas AG1478 did not affect expression of LSR protein in Western blotting (Figure 2D). Treatment with AG1478 inhibited the upregulation of CLDN-2 mRNA induced by knockdown of LSR in real-time PCR analysis (Figure 2E). Treatment with AG1478 was found to prevented the upregulation of CLDN-2 protein induced by EGF in Western blotting (Figure 2F).

### Knockdown of LSR induced cell proliferation, cell migration, and cell metabolism in A549 cells

**Figure 3:**
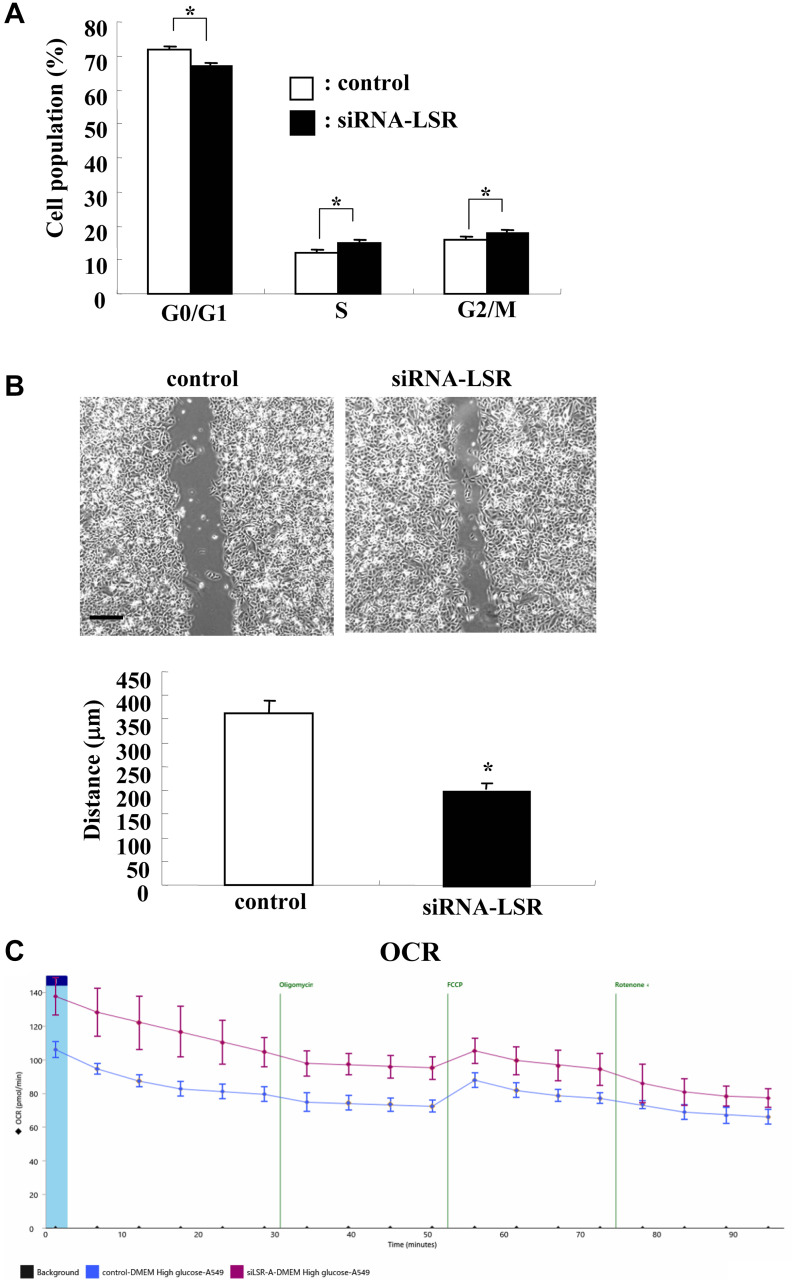
Knockdown of LSR induces cell proliferation, cell migration and OCR in A549 cells. Measurements of cell cycle (**A**), cell migration (**B**), and OCR (**C**) in A549 cells transfected with siRNA-LSR. Scale bar: 400 μm. The distance of (B) is shown as bar graph. ^*^ *p* < 0.05, vs control.

To investigate whether knockdown of LSR affected cell proliferation, cell migration and the cell metabolism in lung adenocarcinoma, we performed knockdown of LSR using the siRNA of LSR and examined the cell cycle, cell migration and cell metabolism. Cell cycle analysis revealed that G0/G1 was significantly decreased and S and G2/M were increased in knockdown of LSR compared to the control (Figure 3A). Knockdown of LSR induced cell migration (Figure 3B). Knockdown of LSR induced aberrant cell metabolism, as measured by the baseline oxygen consumption rates (OCR), maximal OCR, spare respiratory capacity (SRC) and ATP production (Figure 3C).

### Knockdown of CLDN-2 inhibited cell proliferation but not cell migration and cell metabolism in A549 cells

**Figure 4:**
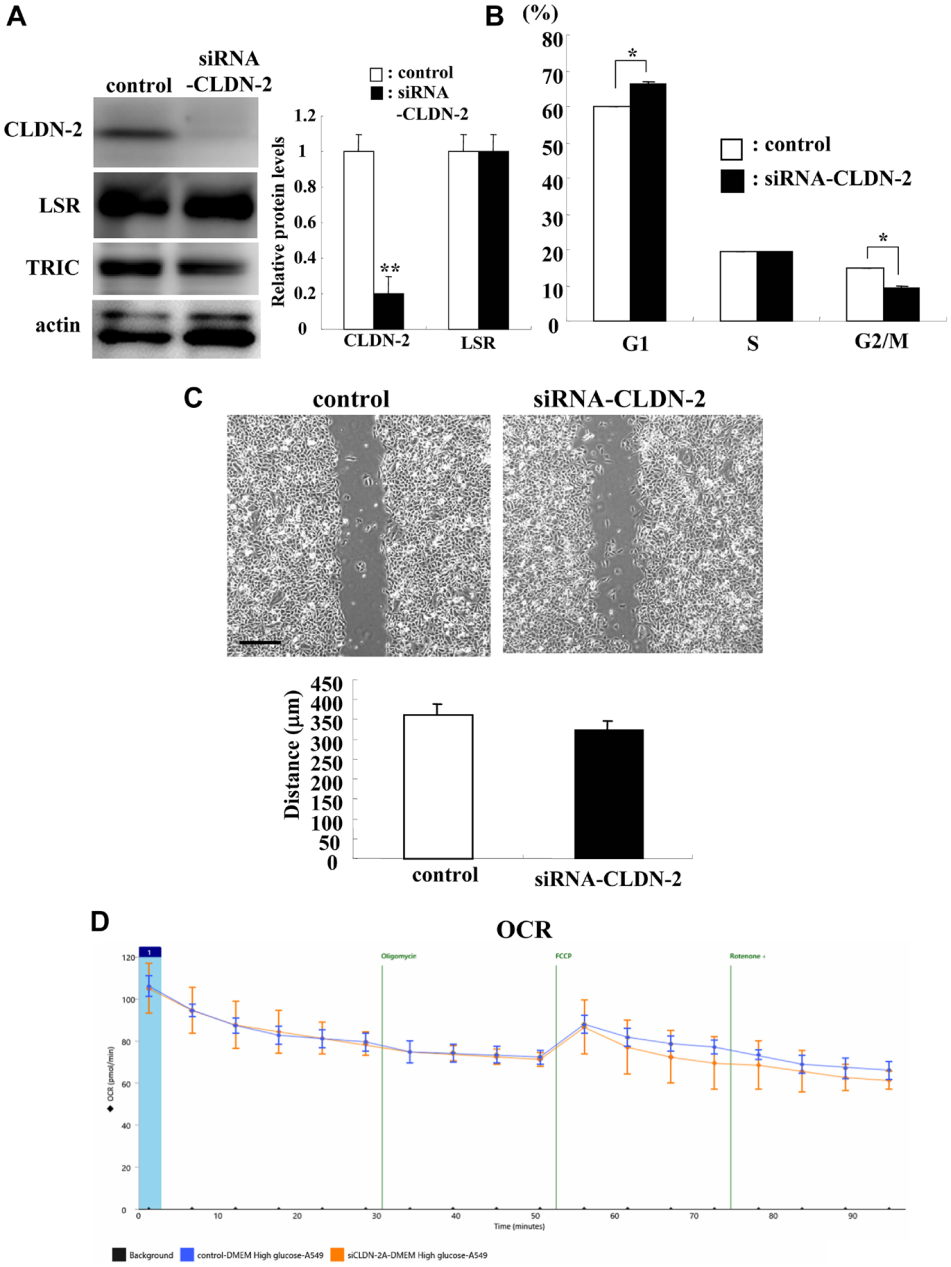
Knockdown of CLDN-2 prevents cell proliferation but not cell migration and OCR in A549 cells. Western blotting (**A**) for LSR, TRIC, and CLDN-2 and measurements of cell cycle (**B**), cell migration (**C**), and OCR (**D**) of A549 cells transfected with siRNA-CLDN-2. Scale bar: 400 μm. The corresponding expression levels of (B) are shown as bar graphs. The distance of (C) is shown as a bar graph. ^*^ *p* < 0.05, vs control.

To investigate whether knockdown of CLDN-2 affected cell proliferation, cell migration and cell metabolism in lung adenocarcinoma, we performed knockdown of CLDN-2 using the siRNA of CLDN-2 and examined the cell cycle, cell migration and cell metabolism. In Western blotting, knockdown of CLDN-2 protein did not affect expression of LSR protein (Figure 4A). In the cell cycle analysis, G0/G1 was significantly increased and G2/M was decreased by knockdown of CLDN-2 compared to the control (Figure 4B). Knockdown of CLDN-2 did not affect cell migration or the cell metabolism (Figure 4C and 4D).

### TGF-β type I receptor inhibitor EW-7197 prevented downregulation of LSR and CLDN-2 induced by TGF-β1 in A549 cells

**Figure 5:**
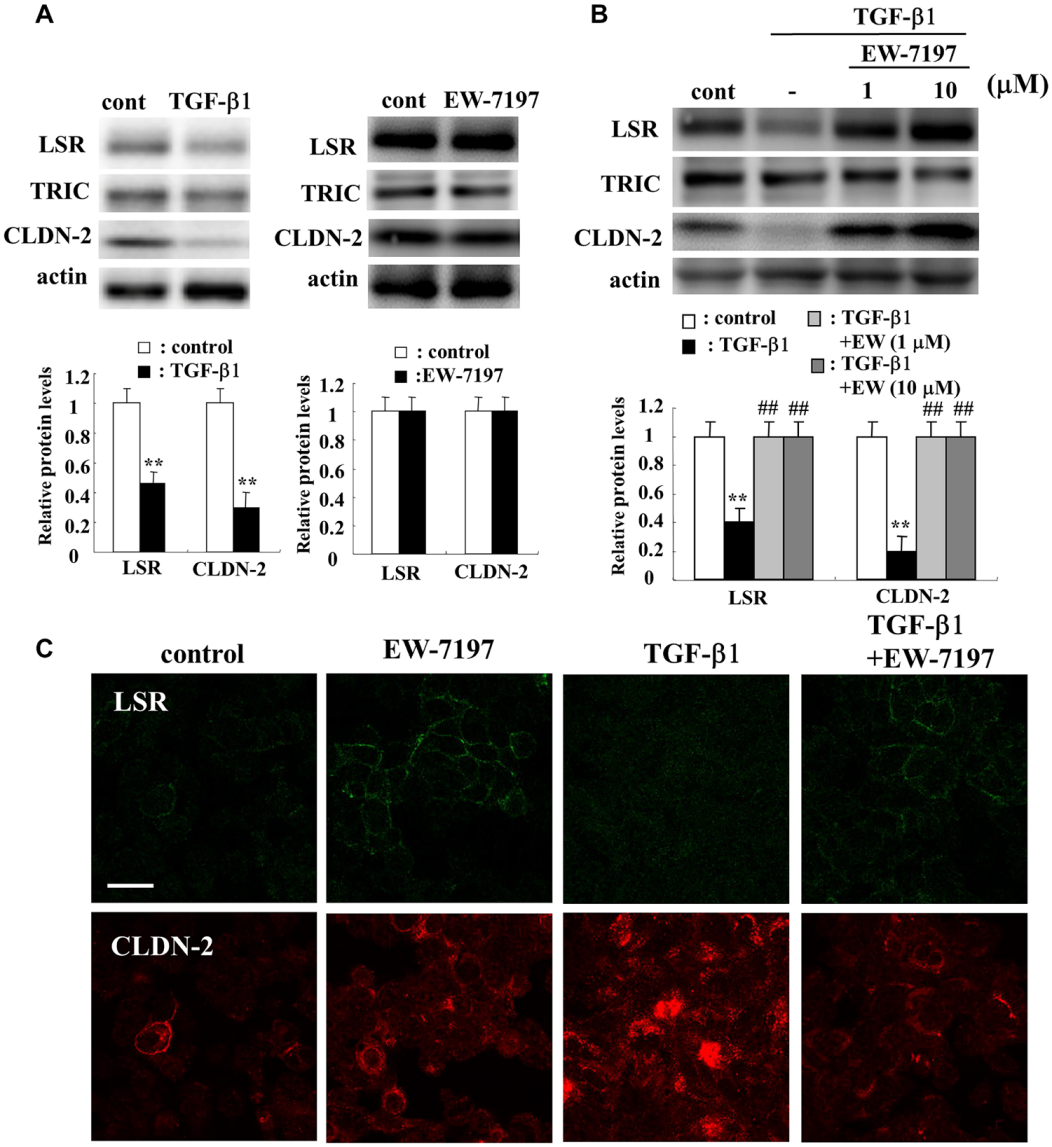
Effects of TGF-β1 and TGF-β receptor inhibitor EW7197 for LSR and CLDN-2 in A549 cells. Western blotting (**A**, **B**) and immunocytochemical staining (**C**) for LSR, TRIC and CLDN-2 in A549 cells treated with 100 ng/ml TGF-β1 with or without 10 μM EW7197. The corresponding expression levels of A and B are shown as a bar graphs. Scale bar: 10 μm. ^**^ *p* < 0.01, vs control, ^#^ *p* < 0.01, vs TGF-β1.

In Western blotting, treatment with TGF-β1 was shown to decreased expression of LSR and CLDN-2 proteins, while treatment with the TGF-β type I receptor inhibitor EW-7197 did not affect them (Figure 5A). EW-7197 prevented the downregulation of LSR and CLDN-2 proteins induced by treatment with TGF-β1 (Figure 5B). Immunostaining showed that EW-7197 induced LSR expression at the membranes of the cells with or without TGF-β1, whereas treatment with TGF-β1 induced CLDN-2 in cytoplasm (Figure 5C).

### EW-7197 prevented upregulation of cell migration and cell metabolism induced by TGF-β1 in A549 cells

**Figure 6:**
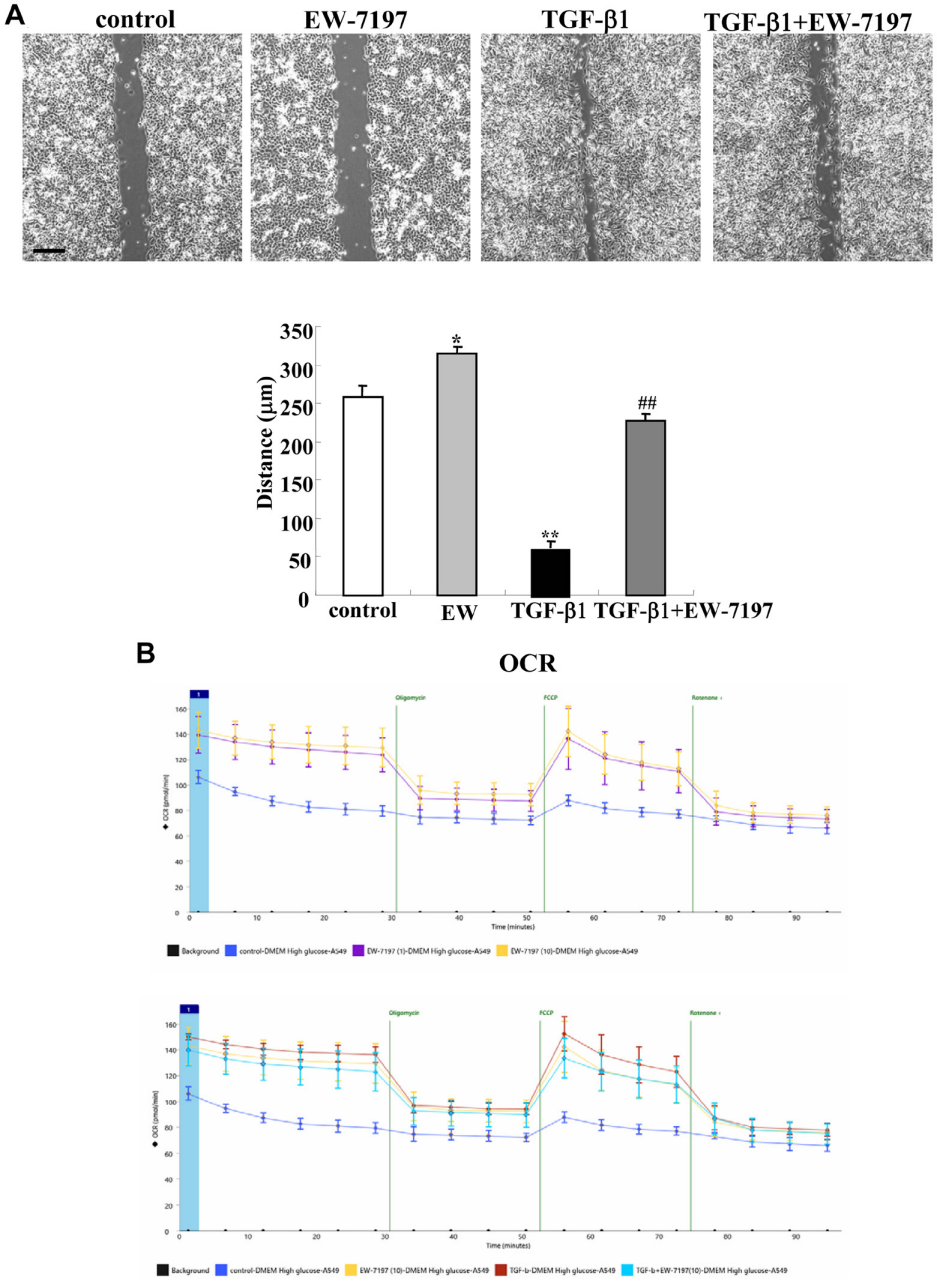
Effects of TGF-β1 and TGF-β receptor inhibitor EW7197 for cell migration and OCR in A549 cells. Measurements of cell migration (**A**) and OCR (**B**) in A549 cells treated with 100 ng/ml TGF-β1 with or without 10 μM EW7197. The distance of (A) is shown as a bar graph. ^**^ *p* < 0.01, vs control, ^*^ *p* < 0.05, vs control, ^##^ *p* < 0.01, vs TGF-β1.

To investigate whether TGF-β1 and EW-7197 affected cell migration and cell metabolism in lung adenocarcinoma, A549 cells were pretreated with EW-7197 before treatment with TGF-β1. Treatment with TGF-β1 induced cell migration and EW-7197 prevented the cell migration in the cells with and without TGF-β1 (Figure 6A). Treatment with EW-7197 or TGF-β1 induced cell aberrant metabolism, as measured by the baseline OCR, maximal OCR, SRC and ATP production (Figure 6B). Pretreatment with EW-7197 prevented the upregulation of cell metabolism induced by TGF-β1 (Figure 6B).

### EW7197 prevented downregulation of LSR and CLDN-2 and upregulation of epithelial permeability induced by TGF-β1 in normal human lung epithelial cells (HLE cells)

**Figure 7:**
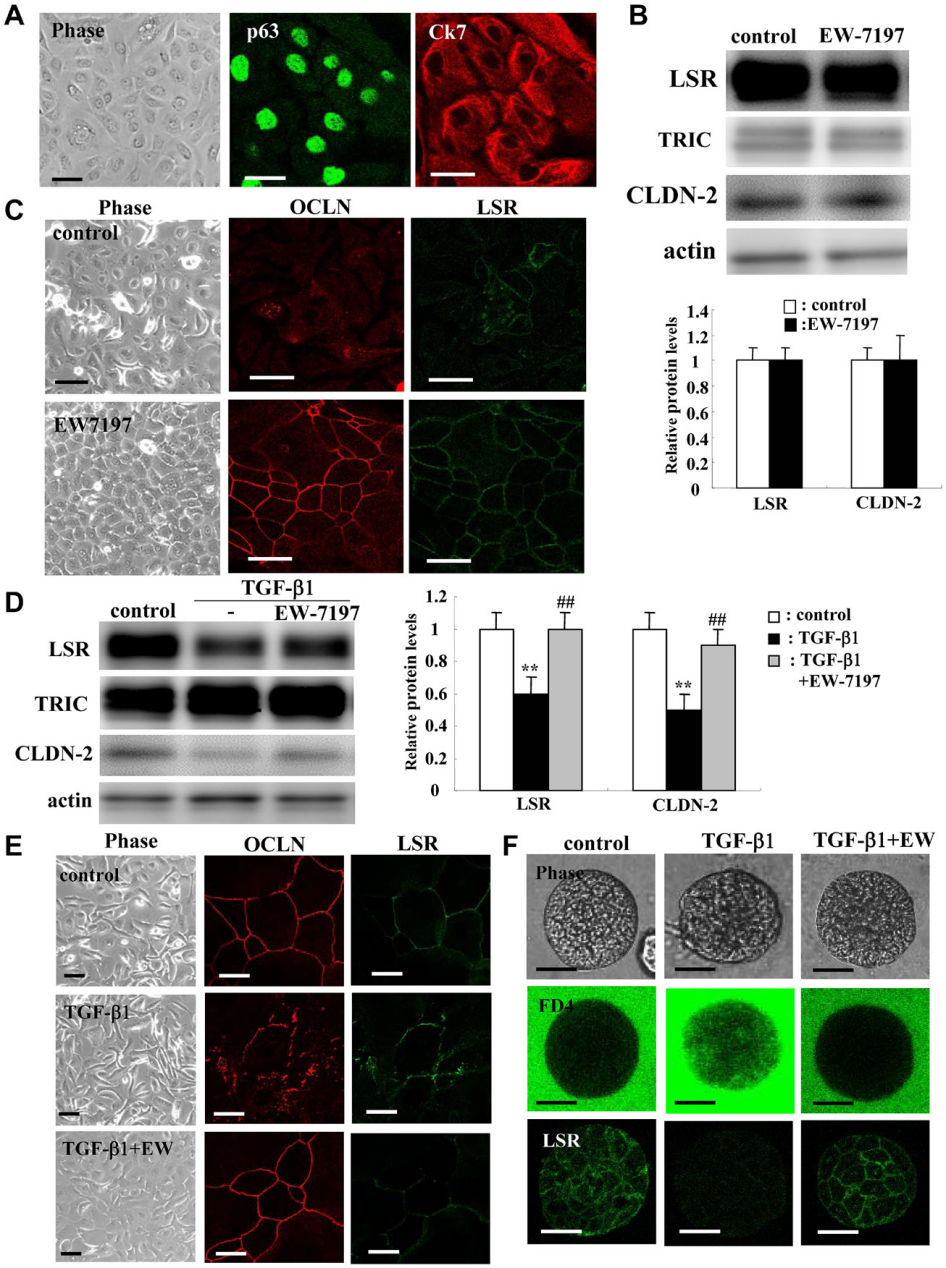
Effects of TGF-β receptor inhibitor EW7197 with and without TGF-β1 for LSR, TRIC, OCLN, and CLDN-2 in human lung epithelial cells (HLE) cells with and without 10% FBS. Immunocytochemical staining (**A**) for p63 and CK7 in HLE cells without FBS. Western blotting (**B**) for LSR, TRIC, and CLDN-2 and immunocytochemical staining (**C**) for LSR and OCLN in HLE cells (without FBS) treated with 10 μM EW7197. Western blotting (**D**) for LSR, TRIC and CLDN-2 and immunocytochemical staining (**E**) for LSR and OCLN in HLE cells (with FBS) treated with 100 ng/ml TGF-β1 with and without 10 μM EW7197. Scale bar: 10 μm. The corresponding expression levels of (B and D) are shown as bar graphs. ^**^ *p* < 0.01, vs control, ^##^ *p* < 0.01, vs TGF-β1. (**F**) Phase contrast, FD-4 permeability assay and immunocytochemical staining for LSR in 2.5D Matrigel-cultured HLE cells (with FBS) treated with 100 ng/ml TGF-β1 with and without 10 μM EW7197. Scale bar: 20 μm.

To investigate whether TGF-β1 and EW-7197 affected normal human lung epithelial cells (HLE cells), HLE cells derived and cultured from lung tissues were treated with TGF-β1 and EW-7197. The basal cell marker p63 and the epithelial marker Ck7 were expressed in all cultured normal HLE cells (Figure 7A). In HLE cells without FBS, EW-7197 markedly induced LSR and the tight junction region marker occluding (OCLN) at the membranes in immunostaining, but it did not affect expression of LSR and CLDN-2 protein (Figure 7B and 7C). In HLE cells with 10% FBS, pretreatment with EW-7197 prevented the downregulation of LSR and CLDN-2 protein, as demonstrated by Western blotting (Figure 7D). Immunostaining showed that treatment with TGF-β1 disrupted OCLN at the membranes, whereas EW-7197 prevented the change (Figure 7E).

TGF-β signaling contributes to the permeability of epithelial cells [28, 41]. Furthermore, analyses of spheroid cells of normal HLE cells are important as those of monolayer cells. To investigate the effects of TGF-β1 and EW-7197 on the epithelial permeability of normal HLE cells, 2.5D Matrigel-cultured HLE cells were treated with FD-4 to measure the permeability. Treatment with TGF-β1 induced permeability of FD-4 and EW-7197 prevented the permeability (Figure 7F). In 2.5D Matrigel-cultured HLE cells, LSR was expressed through the membranes of all cells (Figure 7F). Treatment with TGF-β1 decreased LSR expression and EW-7197 restored the expression (Figure 7F).

## DISCUSSION

**Figure 8:**
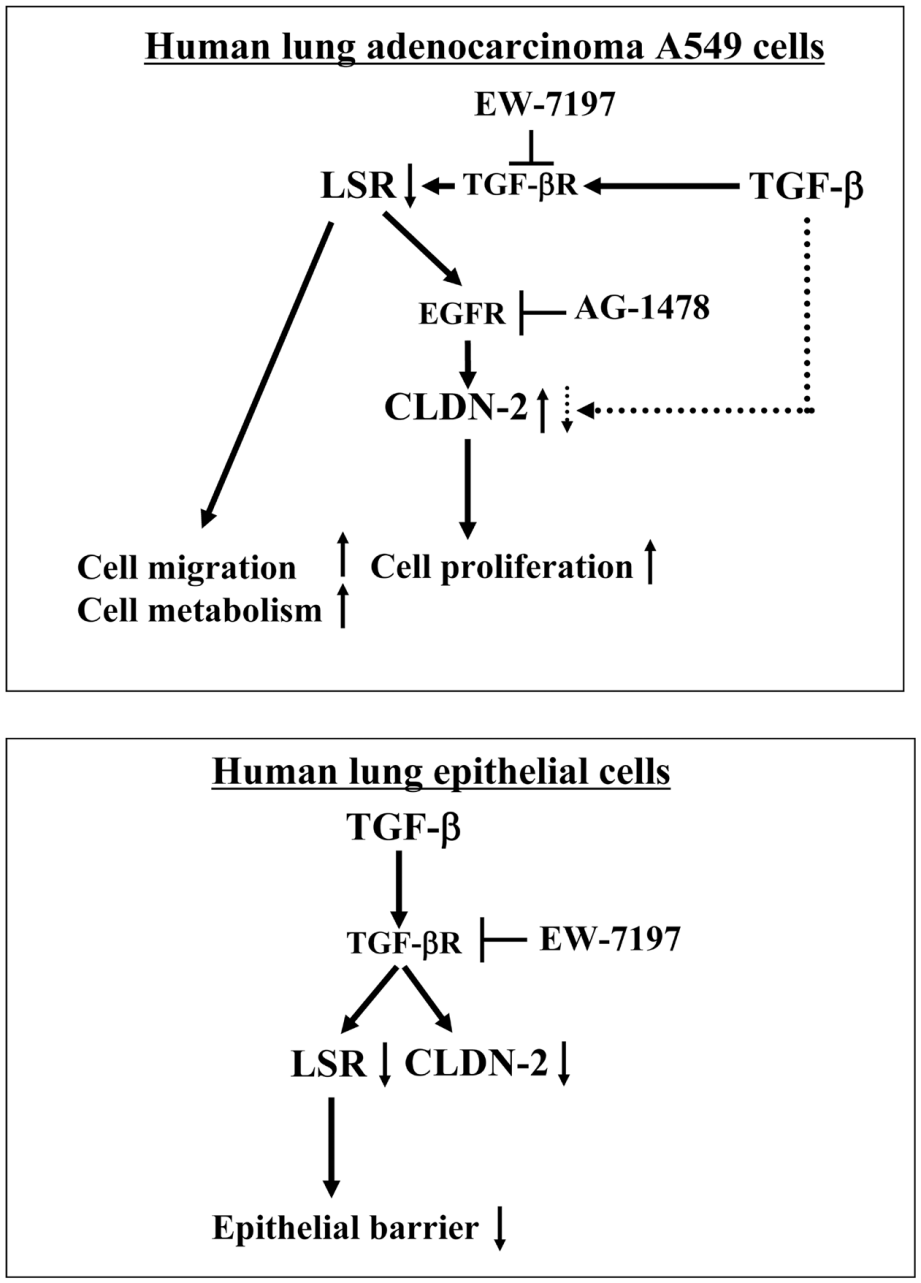
Role and behavior of LSR and CLDN-2 via signaling pathways in human adenocarcinoma A549 cells and normal lung epithelial cells.

In the present study, we found that downregulation of angulin-1/LSR induced malignancy via upregulation of EGF-dependent CLDN-2 and TGF-β-dependent cell metabolism in human lung adenocarcinoma (Figure 8). Loss of angulin-1/LSR affects the malignancy of various cancers, including endometrial cancer, head and neck cancer and pancreatic cancer [17, 18, 20, 21]. In pancreatic cancer, angulin-1/LSR also contributes to the epithelial barrier and malignancy via the growth factors EGF and TGF-β [21]. Knockdown of LSR significantly induces Sp1 transcription factor activity in the CLDN-1 promoter region and promotes cell invasion via CLDN-1-mediated MMPs in endometrial cancer cells [19]. Knockdown of angulin-1/LSR increases the expression of CLDN-2 in airway epithelial Calu-3 cells [14].

CLDN-2 is highly expressed in lung adenocarcinoma tissues and increases proliferation in adenocarcinoma cells [12]. In human lung adenocarcinoma cells, CLDN-2 knockdown decreases matrix metalloproteinase-9 activity and cell migration via suppression of nuclear Sp1 [41]. EGF increases the AP-1 transcription factor activity of CLDN-2 mediated by the activation of an EGFR/MEK/ERK/c-Fos pathway in A549 cells [13, 42].

In the present study, the EGFR tyrosine kinase inhibitor AG1478 prevented the upregulation of CLDN-2 at the protein and mRNA levels induced by EGF in lung adenocarcinoma cell line A549. Knockdown of LSR induced expression of CLDN-2 at the protein and mRNA levels and AG1478 prevented the upregulation of CLDN-2 in A549 cells. Knockdown of LSR induced proliferation and migration of A549 cells. Knockdown of CLDN-2 inhibited the proliferation but did not affect the migration of A549 cells. These findings indicated that knockdown of LSR induced malignancy both dependent on and independent of CLDN-2 expression in lung adenocarcinoma.

Metabolic reprogramming is a hallmark of cancer cells that supports energetic demands for proliferation and invasion of tumor cells [32, 33, 43]. The metabolic reprogramming includes the Warburg effect, indicated as mitochondrial oxidative phosphorylation and high mitochondrial activity, which are important for tumor cell proliferation [43]. TGF-β induces metabolic reprogramming during EMT in cancer [34]. The TGF-β receptor type-1 inhibitor EW-7197 potently inhibits the malignancy of lung adenocarcinoma. However, little is known about TGF-β signaling in lung cancer metabolism.

In the present study, knockdown of LSR, but not CLDN-2, induced aberrant cell metabolism, indicated by the baseline oxygen consumption rate (OCR), maximal OCR, spare respiratory capacity (SRC) and ATP production in A549 cells. Pretreatment with EW-7197 prevented the upregulation of cell metabolism induced by TGF-β1 in A549 cells. These findings suggested that knockdown of LSR induced cell metabolism via TGF-β signaling in lung adenocarcinoma. However, treatment with not only TGF-β1 but also the TGF-β type I receptor inhibitor EW-7197 induced aberrant cell metabolism in A549 cells. The results of treatment with EW-7197 support the existence of the reverse Warburg effect in lung cancer, although the mechanisms are not clear.

TGF-β1 induces not only EMT, but also epithelial permeability in respiratory epithelial cells [28, 44]. In the present study, EW-7197 prevented downregulation of LSR and disruption of OCLN by TGF-β1 in 2D culture of normal human lung epithelial (HLE) cells. It also prevented the upregulation of the epithelial permeability of FD-4 induced by TGF-β1 in 2.5D culture of normal HLE cells. In normal HLE cells without FBS, treatment with TGF-β1 decreased expression of LSR, CLDN-1, -2, -7, and EW-7197 prevented the changes of all tight junction molecules (Figure 7 and Supplementary Figure 2). Furthermore, to investigate whether knockdown of LSR affected the other tight junction proteins in normal HLE cells, the cells were transfected with siRNA of LSR with and without AG1478. In western blotting revealed that knockdown of LSR did not affect TRIC and CLDN-2 in HLE cells with or without AG1478 (Supplementary Figure 3A). In immunostaining, knockdown of LSR did not affect OCLN at the membranes (Supplementary Figure 3B). These results suggested that, in normal HLE cells, TGF-β1 induced not only EMT but also epithelial permeability via downregulation of LSR (Figure 8). However, knockdown of LSR did not affect other tight junction molecules in normal HLE cells.

In the present study Immunohistochemistry showed that CLDN-2 expression in all papillary and invasive lung adenocarcinoma tissues was higher than in normal lung tissues, whereas angulin-1/LSR was highly expressed only in one papillary adenocarcinoma and faintly expressed in the other cases. It is possible that CLDN-2 may be highly expressed in the carcinogenesis of lung adenocarcinoma and LSR may be highly expressed in well-differentiated cancers and then may decrease in poorly differentiated cancers.

In conclusion, AG1478 and EW-7197 demonstrated potent *in vitro* anti-lung adenocarcinoma therapeutic activities via LSR/CLDN-2 and the cell metabolism. The use of both AG1478 and EW-7197 may provide a clinical therapeutic approach for lung adenocarcinoma caused by loss of angulin-1/LSR.

## MATERIALS AND METHODS

### Ethics statement

The protocol for human study was reviewed and approved by the ethics committee of the Sapporo Medical University School of Medicine (Japan). Written informed consent was obtained from each patient who participated in the investigation. All experiments were carried out in accordance with the approved guidelines and with the Declaration of Helsinki (Finland).

### Reagents and antibodies

Rabbit polyclonal anti-tricellulin (TRIC), anti-claudin (CLDN)-1 and -2 antibodies and mouse monoclonal CLDN-2 and anti-occludin (OCLN) antibodies were obtained from Zymed Laboratories (San Francisco, CA, USA). Rabbit polyclonal anti-lipolysis-stimulated lipoprotein receptor (LSR) antibodies were from Novus Biologicals (Littleton, CO, USA). A mouse monoclonal anti-LSR antibody was obtained from Abnova (Taipei, Taiwan). A rabbit polyclonal anti-p63 antibody was obtained from Abcam (Cambridge, UK). Mouse monoclonal anti-acetylated tubulin and antiCK7 antibodies and a rabbit polyclonal anti-actin antibody were from Sigma-Aldrich (St. Louis, MO, USA). Alexa 488 (green)-conjugated anti-rabbit IgG and Alexa 594 (red)-conjugated anti-mouse IgG antibodies and Alexa 594 (red)-conjugated phalloidin were from Molecular Probes, Inc. (Eugene, OR, USA). HRP-conjugated polyclonal goat anti-rabbit IgG was from Dako A/S (Glostrup, Denmark). The ECL Western blotting system was from GE Healthcare UK, Ltd. (Buckinghamshire, UK). A rabbit polyclonal anti-actin antibody and EGFR inhibitor (AG1478) were obtained from Sigma-Aldrich (St. Louis, MO, USA). A TGF-β receptor type 1 inhibitor (EW-7197) was obtained from Cayman Chemical (Ann Arbor, MI, USA). FITC-dextran (FD-4, MW 4.0 kDa) was obtained from Sigma-Aldrich Co. (St. Louis, MO, USA).

### Immunohistochemical analysis

Human lung tissues were obtained from 8 patients with lung adenocarcinoma (2 invasive, 2 papillary, 2 acinar, and 2 solid) who underwent inferior turbinectomy at Sapporo Medical University. Informed consent was obtained from all patients and this study was approved by the ethics committees of the institution. The tissues were embedded in paraffin after fixation with 10% formalin in PBS. Briefly, 5-μm-thick sections were dewaxed in xylene, rehydrated in ethanol, and heated with Vision BioSystems Bond Max using ER2 solution (Leica) in an autoclave for antigen retrieval. Endogenous peroxidase was blocked by incubation with 3% hydrogen peroxide in methanol for 10 min. The tissue sections were then washed twice with Tris-buffered saline (TBS) and preblocked with Block Ace for 1 h. After washing with TBS, the sections were incubated with anti-LSR (1:100) and anti-CLDN-2 (1:400) antibodies for 1 h. The sections were then washed three times in TBS and incubated with Vision BioSystems Bond Polymer Refine Detection kit DS9800. After three washes in TBS, a diamino-benzidine tetrahydrochloride working solution was applied. Finally, the sections were counterstained with hematoxylin. A negative control was performed by replacing the first antibodies with normal rabbit serum. The extent of the positively stained area was scored from 0 to 3 according to the percentage of atypical cells showing positive IHC staining in the observed area: 0 (0%), 1 (1–25%), 2 (26–50%), and 3 (51–100%). The intensity of staining was graded 0 to 3 according to the intensity of immunohistochemical staining: 0 (none), 1 (low), 2 (middle), and 3 (high).

### Cell line culture and treatment

A549 cells and Calu-3 cells derived from human lung adenocarcinomas were purchased from RIKEN Bio-Resource Center (Tsukuba, Japan) and American Type Culture Collection (ATTC, Rockville, MD, USA). A549 cells and Calu-3 cells were maintained with Dulbecco’s modified Eagle’s medium (DMEM) and minimum essential medium (MEM) (Nacalai Tesque, Inc.; Kyoto, Japan) supplemented with 10% dialyzed fetal bovine serum (FBS; Invitrogen, Carlsbad, CA, USA). These medium contained 100 U/ml penicillin, 100 μg/ml streptomycin and 50 μg/ml Amphotericin-B. Sawano cells were plated on 35- and 60-mm culture dishes, that were coated with rat tail collagen (500 μg dried tendon/ml in 0.1% acetic acid), and incubated in a humidified 5% CO_2_ incubator at 37°C. Some cells were treated with 100 ng/ml TGF-β1, 10 μM EW-7197, or 10 μM AG14781.

### Isolation and culture of human lung epithelial (HLE) cells

Human lung tissues were obtained from patients with adenocarcinoma who underwent lobectomy in the Sapporo Medical University Hospital (Japan). Informed consent was obtained from all patients and the study was approved by the ethics committee of Sapporo Medical University.

The human lung tissues were minced into pieces 2 to 3 mm^3^ in volume and washed with PBS containing 100 U/ml penicillin and 100 mg/ml streptomycin (Lonza Walkersville, Walkersville, MD, USA) three times. These minced tissues were digested in 10 ml of Hanks’ balanced salt solution with 0.5 μg/ml DNase I and 0.04 mg/ml Liberase (Roche, Basel, Switzerland) and then incubated with bubbling of mixed O_2_ gas containg 5.2% CO_2_ at 37°C for 20–30 min. The dissociated tissues were subsequently filtered with 300-μm mesh followed by filtration with 40-μm mesh (Cell Strainer, BD Biosciences, San Jose, CA, USA). Stromal cells were removed by filtration, and the remaining cells were backwashed and collected as epithelial cells. After centrifugation at 1000g for 2 min, isolated cells were cultured in bronchial epithelial basal medium (BEBM, Lonza Walkersville MD, USA) containing 4% fetal bovine serum (FBS) (CCB, Nichirei Bioscience, Tokyo, Japan) and supplemented with BEGM^®^ SingleQuots^®^ (Lonza Walkersville MD, USA, including 0.4% bovine pituitary extract, 0.1% insulin, 0.1% hydrocortisone, 0.1% gentamicin, amphotericin-B [GA-1000], 0.1% retinoic acid, 0.1% transferrin, 0.1% triiodothyronine, 0.1% epinephrine, 0.1% human epidermal growth factor), 100 U/ml penicillin, 100 mg/ml streptomycin and 50 μg/ml amphotericin-B on 35- and 60-mm culture dishes (Corning Glass Works, Corning, NY, USA) or in 35-μm glass-wells (Iwaki, Chiba, Japan), coated with rat tail collagen (500 μg of dried tendon/ml of 0.1% acetic acid). The cells were incubated with or without 10% FBS in a humidified 5% CO2:95% air incubator at 37°C. Some cells were treated with 100 ng/ml TGF-β1, 10 μM EW-7197, or 10 μM AG14781.

### 2.5 D matrigel culture

Thirty-five-mm culture glass-coated dishes were coated with 100% Matrigel (15 μl) at 4°C and incubated at 37°C for 30 min. HLE cells (5 × 10^4^) were plated in BEGM medium with 10% Matrigel and cultured for 4 days in the medium without FBS. Some cells were pretreated with 10 μM EW-7197 before treatment with 100 nM TGF-β1.

### RNA interference and transfection

An siRNA duplex oligonucleotide against LSR was synthesized by Thermo Fisher Scientific (Waltham, MA, USA); an siRNA duplex oligonucleotide against CLDN-2 was synthesized by Santa Cruz Biotechnology (Dallas, TX, USA). The sequences were as follows: siRNA of LSR (sense: 5′-CCCACGCAACCCAUCGUCAUCUGGA-3′; antisense: 5′-UCCAGAUGACGAUGGGUUGCGUGGG-3′), siRNA of CLDN-2 (sc37457, sense: 5′-CUAGAAGCCUUACGAAAGAtt-3′; antisense: 5′-UCUUUCGUAAGGCUUCUAGtt-3′). At 24 h after plating, the cells were transfected with 100 nM siRNAs of LSR or CLDN-2 using Lipofectamine™ RNAiMAX Reagent (Invitrogen) for 48 h. A scrambled siRNA sequence (BLOCK-iT Alexa Fluor Fluorescent, Invitrogen) was employed as control siRNA.

### Immunocytochemical staining

A549 cells and HLE cells in 35-mm glass-coated wells (Iwaki, Chiba, Japan), were fixed with cold acetone and ethanol (1:1) at –20°C for 10 min. After rinsing in PBS, the cells were incubated with anti-LSR, anti-CLDN-2, anti-OCLN, anti-p63, and anti-CK7 antibodies (1:100) overnight at 4°C. Alexa Fluor 488 (green)-conjugated anti-rabbit IgG and Alexa Fluor 592 (red)-conjugated anti-mouse IgG (Invitrogen) were used as secondary antibodies. The specimens were examined and photographed with an Olympus IX 71 inverted microscope (Olympus Corp, Tokyo, Japan) and a confocal laser scanning microscope (LSM510; Carl Zeiss, Jena, Germany).

### RNA isolation, RT-PCR, and real-time PCR analysis

Total RNA was extracted and purified using TRIzol (Invitrogen, Carlsbad, CA, USA), and 1 μg was reverse-transcribed into cDNA using a mixture of oligo (dT) and Superscript IV reverse transcriptase according to the manufacturer’s recommendations (Invitrogen). Synthesis of each cDNA was first performed by incubation for 5 min at 65°C and terminated by incubation for 10 min at 80°C in a total volume 20 μl. The polymerase chain reaction (PCR) was performed in a 20-μl mixture containing 100 pM primer pairs, 1.0 μl of the 20-μl total reverse transcription (RT) product, PCR buffer, dNTPs and Taq DNA polymerase according to the manufacturer’s recommendations (Takara, Kyoto, Japan). Amplifications were carried out for 25–40 cycles depending on the PCR primer pair with cycle times of 15 s at 96°C, 30 s at 55°C and 60 s at 72°C. The final elongation time was 7 min at 72°C. Of the total 20-μl PCR product, 7 μl was analyzed by 1% agarose gel electrophoresis with ethidium bromide staining and standardized using a GeneRuler 100-bp DNA ladder (Fermentas, Ontario, Canada). The PCR primers used for LSR, CLDN-2 and glucose-3-phosphate dehydrogenase (G3PDH) by RT-PCR had the following sequences: LSR (sense 5′-CAGGACCTCAGAAGCCCCTGA-3′; antisense 5′-AACAGCACTTGTCTGGGCAGC-3′), CLDN-2 (sense: 5′-CAAATGCTGCTCATGGAAAGA-3′; antisense: 5′-CAGGACCCAGAGGTGTAGGA-3′), and G3PDH (sense 5′-ACCACAGTCCATGCCATCAC-3′; antisense 5′-TCCACCACCCTGTTGCTGTA-3′). Real-time PCR detection was performed using a TaqMan Gene Expression Assay kit with a StepOnePlus™ real-time PCR system (Applied Biosystems, Foster City, CA, USA). The amount of 18S ribosomal RNA (rRNA) (Hs99999901) mRNA in each sample was used to standardize the quantity of the mRNAs of LSR (Hs017067321) and CLDN-2 (Hs00252666). The relative mRNA-expression levels of the control and treated samples were calculated by the difference of the threshold cycle (comparative C_T_ [ΔΔC_T_] method) and presented as the average of triplicate experiments with a 95% confidence interval.

### Western blot analysis

The cultured cells were scraped from 60 mm dishes containing 400 μl of buffer (1 mM NaHCO^3^ and 2 mM phenylmethylsulfonyl fluoride), collected in microcentrifuge tubes, and then sonicated for 10s. The protein concentrations of the samples were determined using a BCA protein assay regent kit (Pierce Chemical Co.; Rockford, IL, USA). Aliquots of 15 μl of 20 μg protein/lane for each sample were separated by electrophoresis in 5–20% SDS polyacrylamide gels (Wako, Osaka, Japan), and electrophoretically transfered to a nitrocellulose membrane (Immobilon; Millipore Co.; Bedford, UK). The membrane was saturated with blocking buffer (25 mM Tris, pH 8.0, 125 mM NaCl, 0.1% Tween 20, and 4% skim milk) for 30 min at room temperature and incubated with anti-LSR, anti-TRIC, anti-CLDN-1, -2, anti-Ac-tubulin and anti-actin antibodies (1:1000) at room temperature overnight. Then it was incubated with HRP-conjugated anti-mouse and anti-rabbit IgG antibodies at room temperature for 1 h. The immunoreactive bands were detected using an ECL Western blotting system.

### Migration assay

After the A549 cells were plated onto the 35 mm dishes, they were cultured to confluence. At 24 h we wounded the cell layer using a plastic pipette tip (P200), and measured the length of the wound by using a microscope imaging system (Olympus, Tokyo, Japan).

### XF96 extracellular flux measurements

Mitochondrial respiration was assessed using an XF96 Extracellular Flux Analyzer (Aligent, Santa Clara, CA, USA; Supplementary Figure 4). A549 cells were seeded on XF96 plates at a density of 20,000 cells/well after incubation in DMEM medium with high glucose or glucose-free medium for 24 h. One day prior to the experiment, sensor cartridges were hydrated with XF calibrate solution (pH 7.4) and incubated at 37°C in a non-CO_2_ incubator for 24 h. Baseline measurements of mitochondrial respiration (OCR) were taken before sequential injection of the following inhibitors: 1 μM oligomycin, which is an ATP synthase inhibitor; 2 μM FCCP, which is a mitochondrial respiration uncoupler; and 1 μM antimycin A and rotenone, which are mitochondrial electron transport blockers. Oligomycin was applied first to estimate the proportion of basal OCR coupled to ATP synthesis. After oligomycin application, FCCP was used to further determine the maximal glycolysis pathway capacity.

### Fluorescein isothiocyanate (FITC) permeability assay

To assess barrier function, the permeability of Fluorescein isothiocyanate (FITC)-dextran (FD-4, MW 4.0 kDa) from the outside into the spheroid lumen was examined by using 2.5D Matrigel culture of HLE cells on 35-mm glass-coated dishes. 2.5D Matrigel culture of HLE cells were incubated in the medium with 1% FD-4 at 37°C for 2 h. Ten spheroids of all experiments were photographed and measured by a confocal laser scanning microscope with imaging soft (LSM5 PASCAL; Carl Zeiss, Jena, Germany).

### Data analysis

Signals were quantified using Scion Image Beta 4.02 Win (Scion, Frederick, MD, USA). Each set of results shown is representative of at least three separate experiments. Results are given as means ± SEM. Statistical analysis was tested by one-way analysis of variance (ANOVA) with the Tukey–Kramer method.

## SUPPLEMENTARY MATERIALS


