# Supplementary material for: Downregulation of angulin-1/LSR induces malignancy via upregulation of EGF-dependent claudin-2 and TGF-β-dependent cell metabolism in human lung adenocarcinoma A549 cells
Source: Oncotarget. 2023 Mar 24;14:261–75. doi: 10.18632/oncotarget.27728 (PMC10038356; doi:10.18632/oncotarget.27728)
Supplement: Supplementary file 1 [file oncotarget-14-27728-s001.pdf]

Downregulation of angulin-1/LSR induces malignancy via upregulation of EGF-dependent claudin-2 and TGF-β-dependent cell metabolism in human lung adenocarcinoma A549 cells

SUPPLEMENTARY MATERIALS

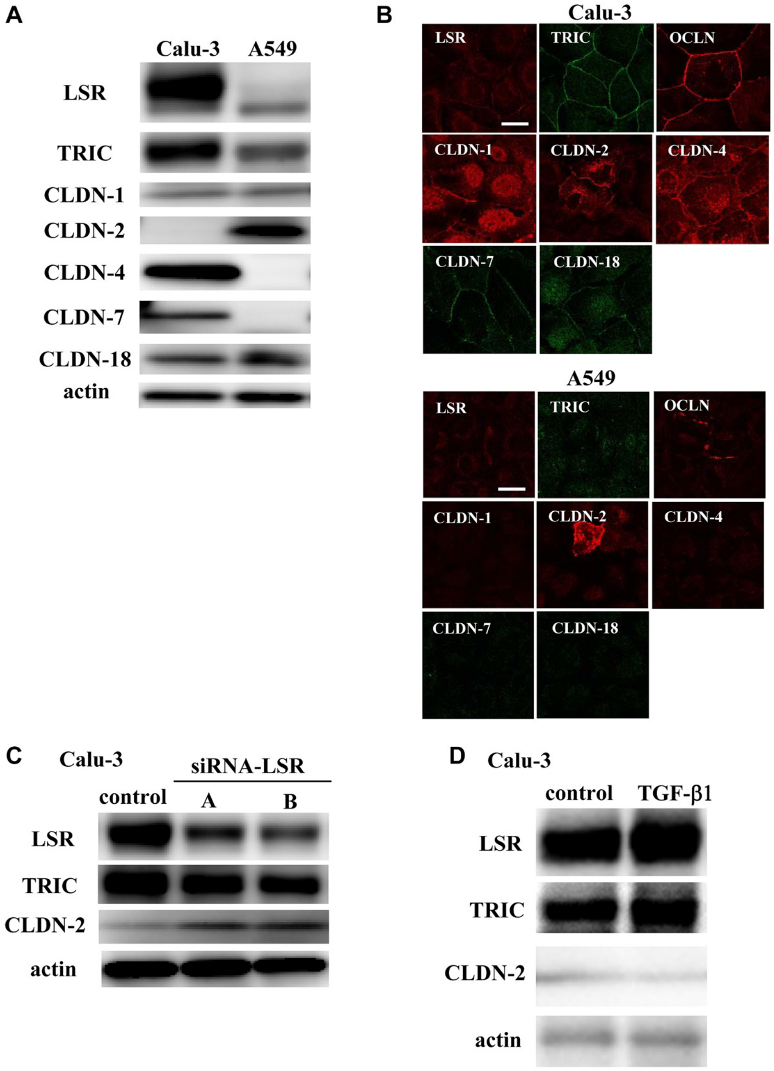

Supplementary Figure 1: Effects of knockdown of LSR and treatment with TGF-β in human lung adenocarcinoma Calu-3 cells. Western blotting (**A**) for tight junction proteins in human lung adenocarcinomas Calu-3 cells and A549 cells. Immunocytochemical staining (**B**) for tight junction proteins in Calu-3 cells and A549 cells. Scale bar: 10 μm. Western blotting (**C**) for LSR, TRIC, and CLDN-2 in Calu-3 cells transfected with siRNA-LSR. Western blotting (**D**) for LSR, TRIC, and CLDN-2 in Calu-3 cells treated with 100 ng/ml TGF-β1.

HLE cells

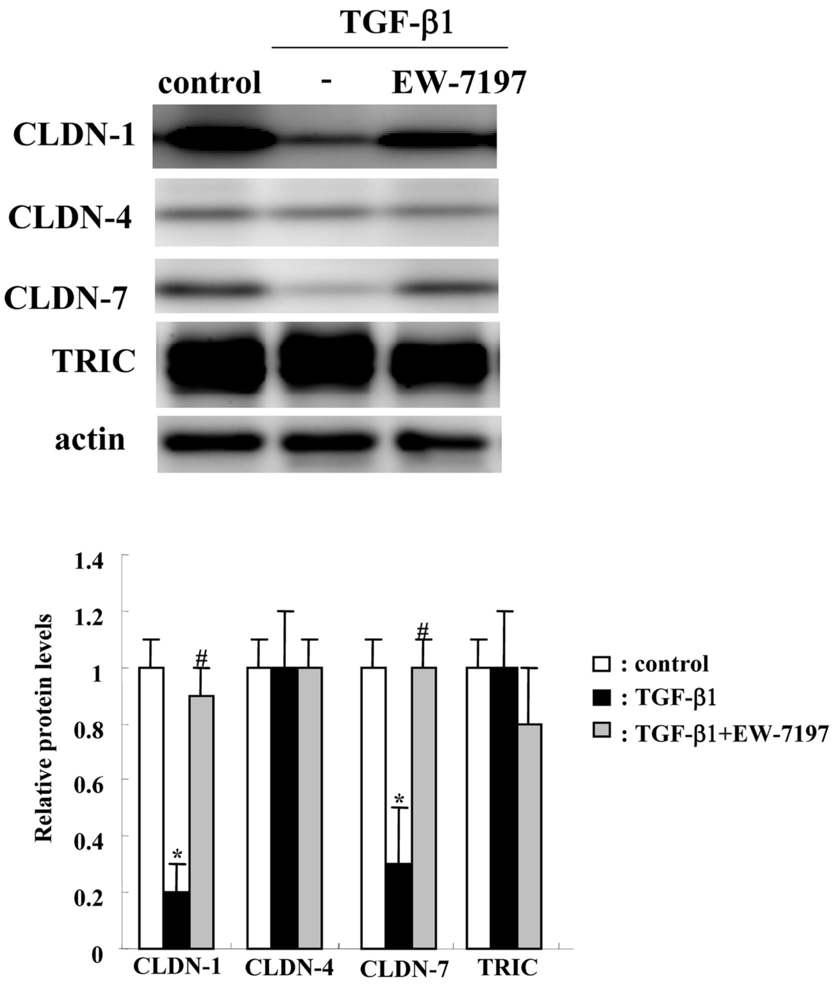

Supplementary Figure 2: Effects of TGF-β1 and TGF-β receptor inhibitor EW7197 for CLDN-1, -4, -7, and TRIC in human lung epithelial cells without 10% FBS . Western blotting for CLDN-1, -4, -7, and TRIC in human lung epithelial cells pretreated with 10 μM EW7197 before treatment with 100 ng/ml TGF-β1. The corresponding expression levels are shown as a bar graph. Scale bar: 10 μm. $^{*}p < 0.05$, vs control, $^{\#}p < 0.05$, vs TGF-β1.

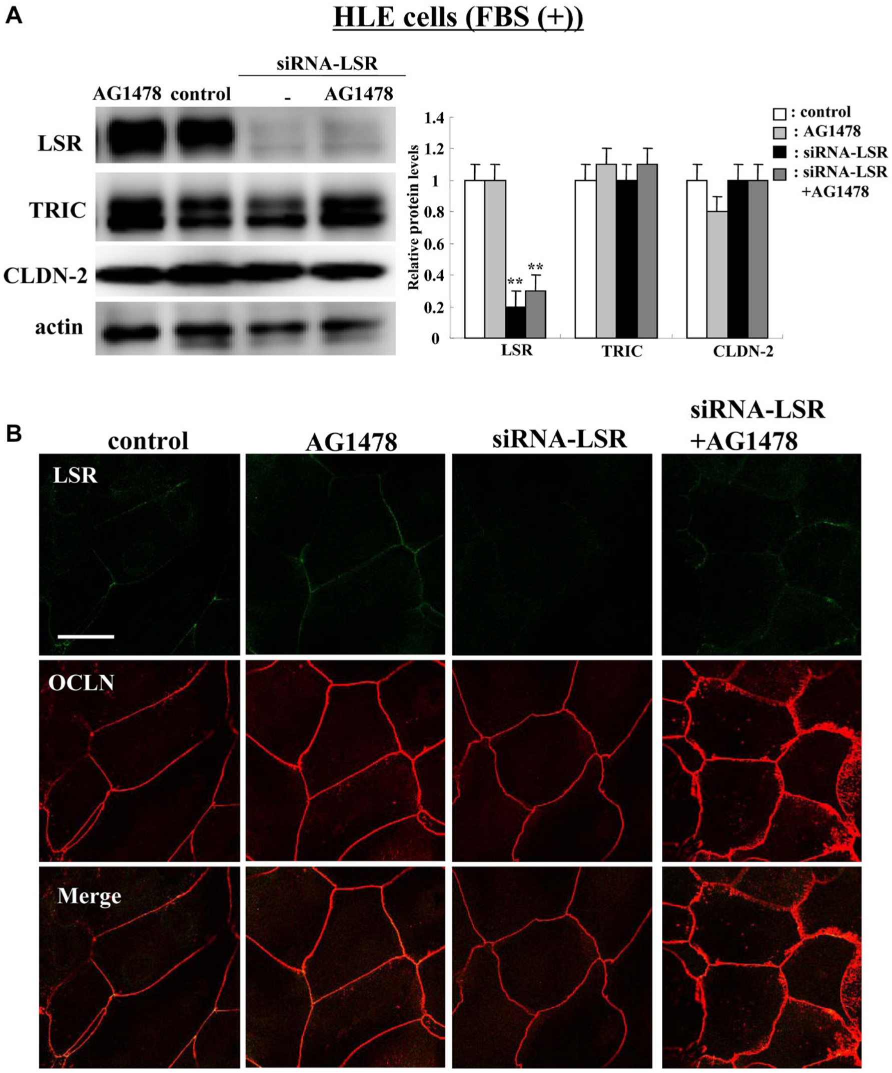

Supplementary Figure 3: Effects of knockdown of LSR in human lung epithelial cells with 10% FBS. Western blotting (**A**) for LSR, TRIC, and CLDN-2 and immunocytochemical staining (**B**) for LSR and OCLN in human lung epithelial cells (with 10% FBS) transfected siRNA of LSR with and without 10 μM EW7197. The corresponding expression levels of A are shown as a bar graph. Scale bar: 10 μm.

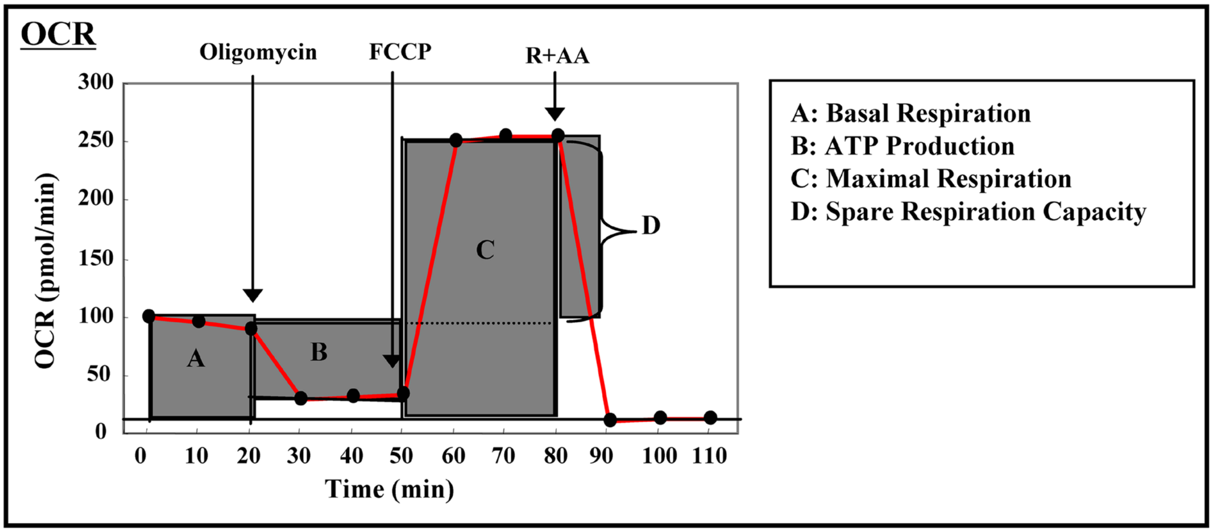

Supplementary Figure 4: Schematic of OCR measurements.
